# Clinical Implications of *HSC70* Expression in Clear Cell Renal Cell Carcinoma

**DOI:** 10.7150/ijms.43100

**Published:** 2021-01-01

**Authors:** Yixiao Zhang, Xudong Zhu, Xinbo Qiao, Lisha Sun, Tianhui Xia, Caigang Liu

**Affiliations:** Department of Oncology, Shengjing Hospital of China Medical University, Shenyang, Liaoning Province, 110004, China.

**Keywords:** clear cell renal cell carcinoma, HSC70, prognosis

## Abstract

**Purpose:** The role of heat shock protein 70 (HSC70) in the progression of clear cell renal cell carcinoma (ccRCC) is unclear. This study explored the effect of the HSC70 on the survival of ccRCC patients.

**Methods:** Immunohistochemical analysis was performed to determine HSC70 expression in samples obtained from 121 ccRCC patients with at least 5 years of follow-up. We also analyzed the association between HSC70 expression and clinicopathological characteristics. Furthermore, the association of overall survival (OS) with HSC70 expression was analyzed using Kaplan-Meier curves. Finally, we used the Oncomine and CCLE databases to determine the effects of HSC70 mRNA expression on ccRCC.

**Results:** HSC70 expression was associated with distant metastasis and death of ccRCC patients. HSC70 was expressed in the nucleus and/or cytoplasm of ccRCC cells. The incidence of distant organ metastasis and death was higher in patients with HSC70 expression than in those without it. Survival analysis revealed that patients with HSC70 expression had significantly shorter OS. Oncomine analyses also showed that the HSC70 mRNA was significantly upregulated in ccRCC tissues.

**Conclusions:** HSC70 expression was related to adverse prognosis, and patients with HSC70 expression had a worse prognosis than those without HSC70 expression. *HSC70* may thus serve as a potential therapeutic target for ccRCC.

## Introduction

Renal carcinoma is one of the most common urological malignancies and accounts for 3% of all tumors 1. The incidence of renal carcinoma has been on the increase worldwide 2. Despite many advances in cancer diagnosis and treatment in recent decades, especially improved imaging techniques and operation procedures, the strategies for treating renal cancer remain limited owing to resistance against chemotherapy and radiotherapy 3. The high probability of tumor recurrence and metastasis results in patient poor outcomes in renal cancer 4. The mechanisms underlying renal cancer metastasis remain unclear. Thus, there is an urgent need to elucidate the molecular mechanisms underlying renal cancer tumorigenesis and to develop novel therapeutic strategies, including treatments aimed at specific molecular targets, to reduce renal cancer-related mortality.

The heat shock protein 70 (HSC70) family represents a group of molecular chaperones that can support protein synthesis and prevent the formation of aggregates 5. HSC70 plays an important role in several processes, such as nascent polypeptide folding, chaperone-mediated autophagy, and protein translocation across membranes. Accumulation of incorrect proteins in cancer cells and deregulated signaling pathways could influence the survival outcomes, and the maintenance of protein homeostasis by HSC70 may be key for the growth and survival of cancer cells 6.

High levels of HSC70 expression are associated with many kinds of cancers, making it a potential therapeutic target. The expression of HSC70 is higher in human prostate cancer cells compared to that in normal cells 7. The expression HSC70 is frequently increased in cancer tissues obtained from patients with colon cancer 8. In the human colon cancer cell line HT29, HSC70 knockdown decreases cell viability and leads to cell death. In 160 breast cancer cases, 97% (155) were found to be positive for HSC70 expression, and the expression is usually stronger than that in adjacent non-cancer cells 9. Although HSC70 is known to be expressed at high levels in cancer cells and tissues, there is a dearth of information regarding the relationship between the HSC70 expression and renal cell carcinoma.

In this study, we investigated the role of HSC70 with respect to the survival outcomes of patients with renal cell carcinoma.

## Methods

### Patients and cancer specimens

We enrolled 121 patients with clear cell renal cell carcinoma. Surgical cancer tissues were collected at the China Medical University between January 2013 and December 2015. All 121 tissue specimens were confirmed pathologically and graded by two independent pathologists, according to the Fuhrman nuclear grade. The inclusion criteria included a histological demonstration of clear cell renal cell carcinoma, 18-80 years of age, absence of distant metastasis at surgery, and follow-up for up to 5 years after surgery. Surgical specimens of the primary renal cell tumors were immunohistochemically stained using the anti-HSC70 antibody. The exclusion criteria included preoperative adjuvant treatment and death unrelated to renal carcinoma. The protocol was approved by the China Medical University Ethics Committee. The clinicopathological characteristics included in the study were age, histological grade, serum Ca^2+^ levels, hemoglobin levels, neutral granulocyte count, and platelet count.

### Immunohistochemistry

Resected renal cancer specimens were fixed in 4% formaldehyde and embedded in paraffin. Cancer tissue samples, which were sliced into 5-μm‒thick sections and placed on glass slides treated with 3-aminopropyltriethoxysilane, were deparaffinized and rehydrated. After that, they were incubated overnight at 4 °C with a primary antibody against HSC70 (1:50) purchased from Abcam (ab51052). After washing, cells probed with the primary HSC70 antibody were detected by incubating with horseradish peroxidase (HRP)-conjugated secondary antibody at 37 °C for 45 min (purchased from Gene Tech, Shanghai), and then with DAB (DAB kit; purchased from Gene Tech, Shanghai). The primary antibody was substituted with phosphate-buffered saline in the negative control. Immunohistochemical signals were evaluated independently by two pathologists.

The expression of HSC70 was semi-quantitatively classified according to the following criteria: a score of 0 if <1% of cancer cells expressed nuclear and/or cytoplasmic HSC70; 1+ if ≥1% and <5% of cancer cells expressed nuclear and/or cytoplasmic HSC70; 2+ if ≥5% and <10% of morphologically unequivocal cancer cells expressed nuclear and/or cytoplasmic HSC70; and 3+ if ≥10% were positive cells; 2+ and 3+ were considered HSC70-positive.

### Statistical analysis

Overall survival (OS) was defined as the time from the date of the surgery to the date of death. Relationships between OS and positive or negative HSC70 expression were evaluated using the independent sample* t*-test. The OS of these renal carcinoma patients was estimated using Kaplan-Meier survival curves, and the difference was analyzed using the log-rank test. All data were analyzed using the SPSS software (version 21.0; SPSS, IL, USA). *P*-values less than 0.05 were considered statistically significant.

### Oncomine analysis

Analysis using the Oncomine database (www.oncomine.org) allowed the determination of HSC70 mRNA levels and DNA copy numbers in different types of cancer. The *t*-test was used to determine *p*-values when comparing cancer tissues with normal control datasets. Multiple change was defined as 2, and the *P*-value was set at 0.01. Significant correlations can be found in a series of studies, which are shown in typical diagrams.

### CCLE analysis

Analysis using the CCLE database allowed the determination of HSC70 mRNA levels in a series of cancers. The number of chromosome copies and information from many edited online encyclopedias were generated from parallel sequencing data from 947 human cancer cell lines to facilitate the identification of genetic, pedigree, and predictive factors of drug sensitivity.

## Results

### HSC70 expression and clinicopathological characteristics of renal cancer patients

One hundred twenty-one surgical renal cancer specimens were analyzed using immunohistochemistry. The basic relationships between HSC70 expression and the clinicopathological characteristics are shown in **Table 1.** HSC70 expression was not significantly related to age, histological grade, serum Ca^2+^ levels, hemoglobin levels, neutral granulocyte count, or platelet count; however, it was associated with distant metastasis, death, and OS (*P* = 0.019).

**Figure 1:**
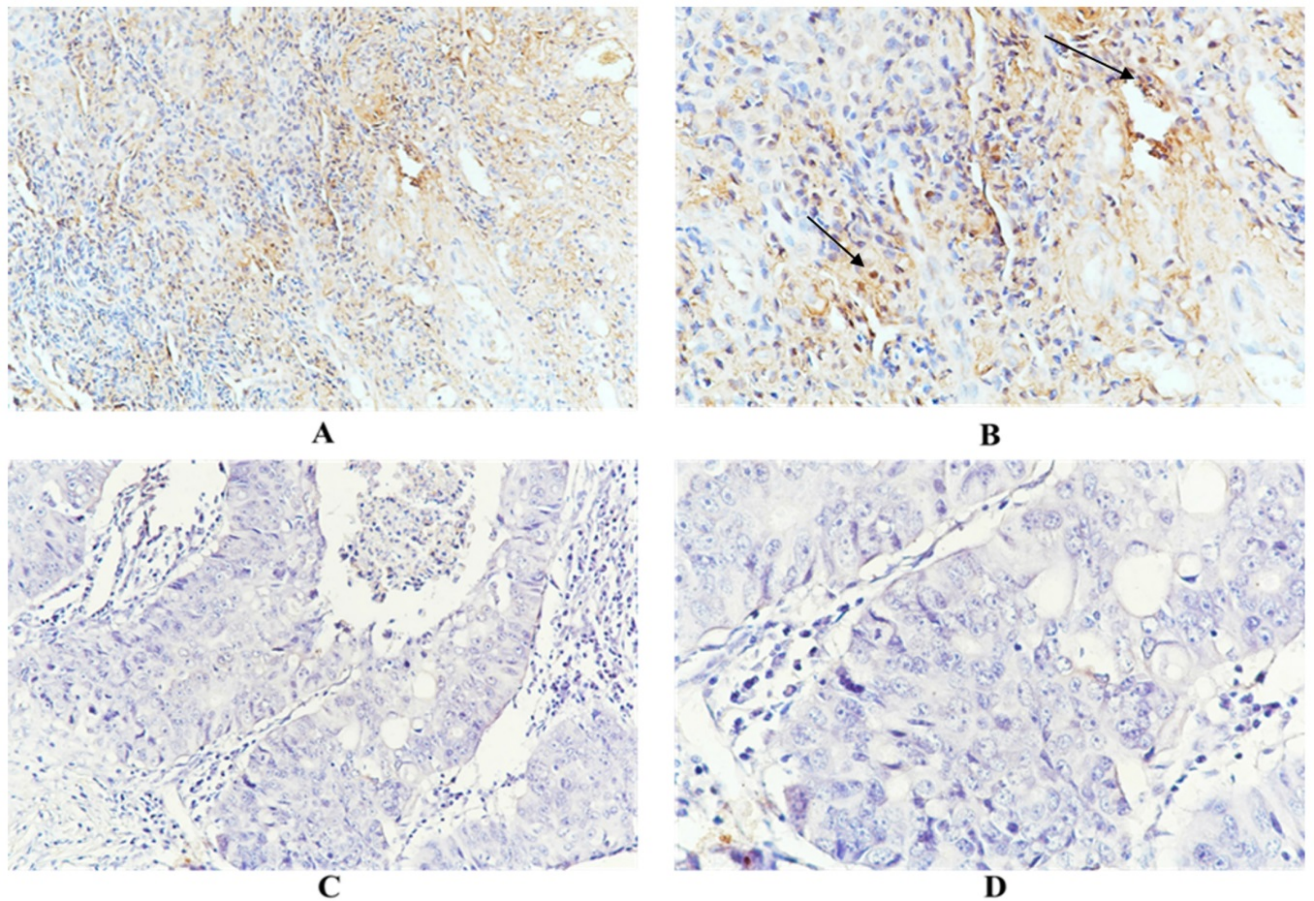
Representative positive HSC70 (A and B. A, x200 magnification. B, x400 magnification) and negative HSC70 immunohistochemical staining (C and D. C, x200 magnification. D, x400 magnification).

Immunohistochemical examination showed that HSC70 was expressed in the nucleus and/or cytoplasm of renal cancer cells, and HSC70 was not detected when the primary antibody was omitted. As shown in **Figure 1**, positive and negative anti-HSC70 staining were observed in tumor samples from renal cancer patients. HSC70 expression was observed in 50 patients, with a positive rate of 41.3%. A total of 22 (18.2%) patients showed distant metastases. Among them, 14 patients were HSC70-positive, with a positivity rate of 63.6%. There were 20 (16.5%) deaths, 13 of the deceased individuals had exhibited HSC70-positive signals (positivity rate, 65%). Overall, renal cancer patients with distant metastases or deceased patients expressed higher levels of HSC70 than patients without distant metastases or those who survived (**Figure 1**).

**Figure 2:**
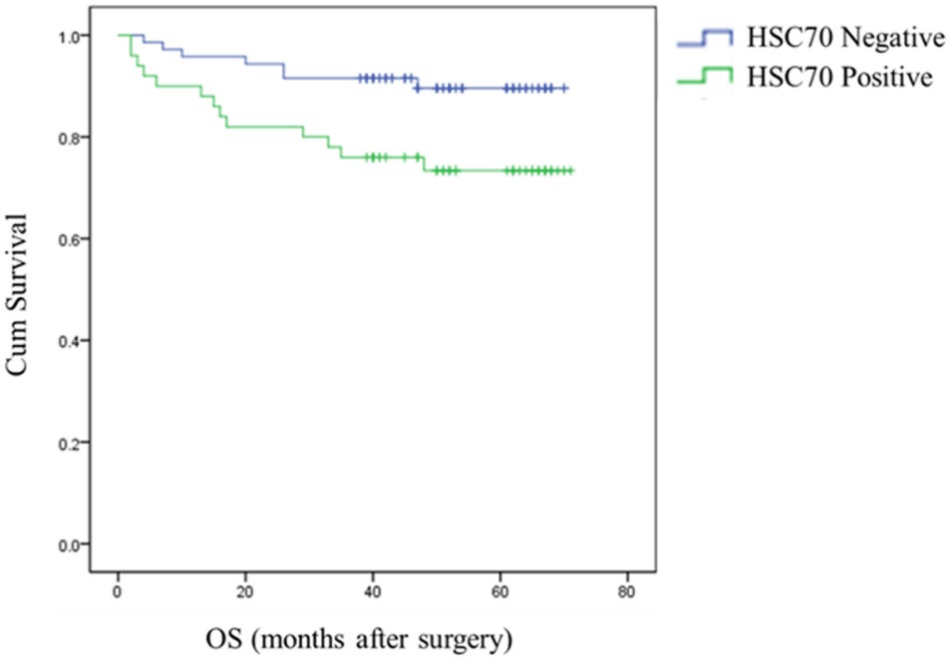
HSC70 expression was associated with reduced OS (*P* = 0.018, log-rank test).

The analysis indicated that HSC70 expression in renal cancer tumors was significantly associated with OS. Survival curves showed that renal cancers patients with positive HSC70 expression had a significantly shorter OS than those with negative HSC70 expression. HSC70 positivity is associated with a shorter OS and poor prognosis (**Figure 2**).

### Analysis of differential HSC70 expression in renal cancer

Using Oncomine, we investigated the differential expression of HSC70 mRNA between different types of renal cancers and normal tissues. The results show that *HSC70* mRNA expression was significantly higher in renal cancer than that in normal samples and different cancer types. The levels of *HSC70* transcripts were elevated 1.562-fold in papillary renal cell carcinoma samples compared to those in normal tissues in a dataset containing 11 samples derived from the Jones study (*P* = 1.93×10^-7^; **Supplementary Figure 1A**) and elevated 1.413-fold in renal pelvis urothelial carcinoma samples compared to those in normal tissues (*P* = 1.95×10^-4^; **Supplementary Figure 1B**). In a dataset from the Yusenko study containing 67 samples, the levels of *HSC70* were elevated 1.511-fold in renal Wilms tumor samples compared to those in normal tissues (*P* = 5.34×10^-5^; **Supplementary Figure 1C**). In another dataset, the levels of *HSC70* mRNA transcripts were 1.329-fold higher in papillary renal cell carcinoma compared to those in normal tissues (*P* = 9.12×10^-4^; **Supplementary Figure 1D**). In datasets of different cancer types, the copy number of the *HSC70* DNA in kidney cancer was comparable to that in normal tissues. *HSC70* copy number increased only 1.007-fold (*P* = 0.005) in kidney cancer samples compared to that in normal tissues in a dataset of 1071 samples from The Cancer Genome Atlas (TCGA) (**Supplementary Figure 1E**). To obtain a more comprehensive conclusion, we conducted a meta-analysis on multiple datasets, which showed that there is significant differential expression of *HSC70* in renal cancer (**Supplementary Figure 1F**).

Unexpectedly, the results of CCLE analysis differed from those of the Oncomine analysis. *HSC70* was not upregulated in a renal cancer cell line and ranked only 31 in many cell lines (**Supplementary Figure 2**).

### Co-expression analysis of HSC70 in different molecular subtypes of renal carcinoma

**Figure 3:**
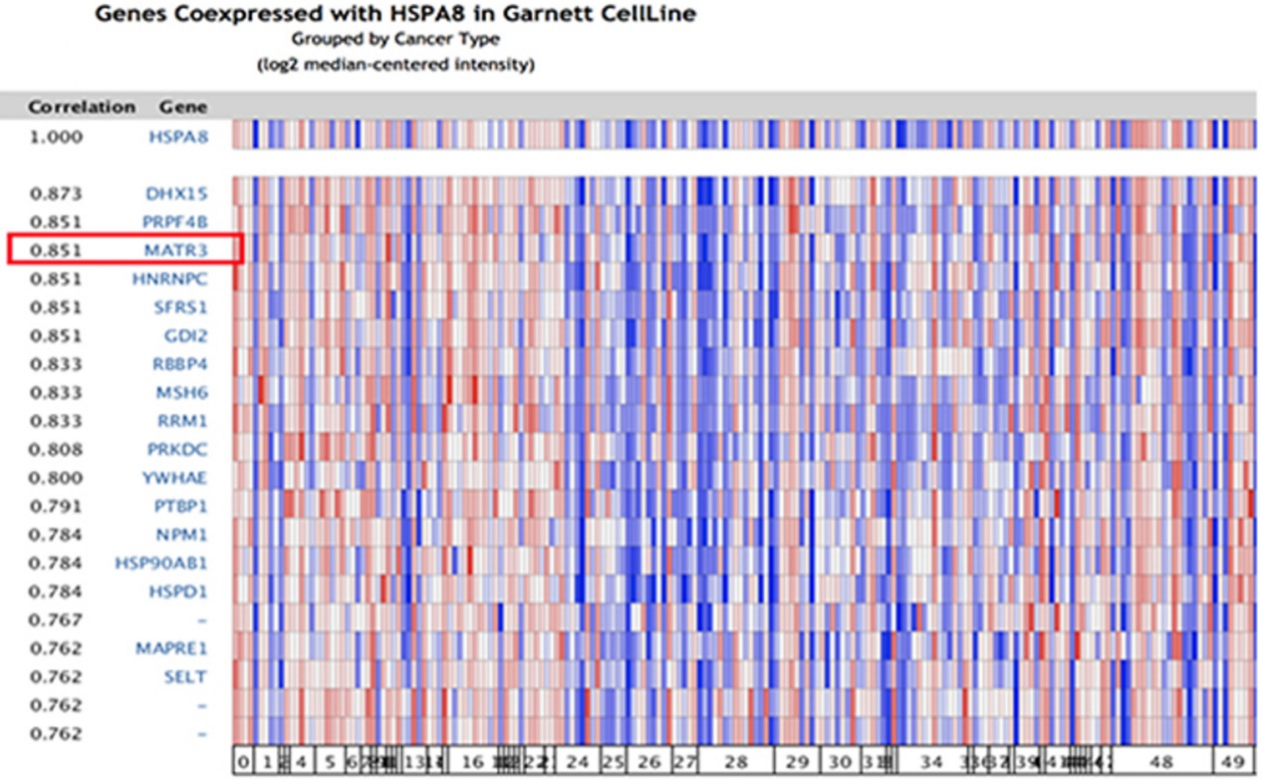
Correlation analysis of HSC70 in different molecular subtypes of renal cancer. In the Oncomine analysis, the expression of *HSC70* is significantly correlated with *MATR3* expression (shown in red box).

Because significant differences were found in HSC70 expression in renal cancer patients, we next explored the potential role of HSC70 in renal cancer and its association with other characteristic biomarkers based on the molecular subtypes. In Oncomine co-expression analysis, *HSC70* expression was found to be significantly correlated with *MATR3* (r = 0.851; **Figure 3**). This result predicted that *HSC70* might function in combination with *MATR3* to regulate the biological behavior of renal carcinoma cells and tissues.

## Discussion

Renal cancer is a clinically heterogeneous disease, and many factors influence its prognosis 10. In the absence of effective therapy, renal cancer can metastasize to other tissues or organs, or recur after therapy, resulting in death. The high probability of tumor recurrence and metastasis results in poor outcomes for patients with renal cancer 11,12. Multiple proteins are involved in the process of cancer metastasis 13-16. At present, the mechanisms underlying renal cancer metastasis remain unclear. Distant metastatic renal carcinoma is usually difficult to treat, and patients with this condition usually have a short OS, highlighting the importance of finding new and effective indicators of prognosis for renal cancer patients.

HSC70 maintains protein homeostasis in both normal and stress conditions. HSC70 can also regulate the translocation of proteins into cellular organelles 17,18. To our knowledge, no clinical studies have explored the association between HSC70 expression and the survival outcomes of renal cancer patients.

In our study, we used immunohistochemistry to examine HSC70 expression in 121 surgical renal cancer tumors. HSC70 expression was observed in 50 patients, with a positivity rate of 41.3%. A total of 22 patients exhibited distant metastases, 14 of whom exhibited the expression of HSC70, with a positivity rate of 63.6%. A total of 22 patients died, 13 of the deceased patients exhibited the expression of HSC70, with a positivity rate of 65%. Higher expression of HSC70 in these patients was related to metastases, death, shorter OS, and poor prognosis. These results suggest that HSC70 may play a key role in the progression of renal carcinoma. To our knowledge, this research may be the first report to explore the expression of HSC70 in renal cancer tumors. Further analysis indicated that patients with HSC70-negative renal cancer had a significantly higher distant metastasis rate, significantly higher death rate, shorter OS, and poorer prognosis compared to those with HSC70-negative renal cancer. We believe that HSC70 expression is an independent risk factor for higher distant metastasis rates, higher death rates, and shorter OS. We showed that in the dataset with 489 renal cancer samples, a clear difference was observed in HSC70 copy numbers between renal cancer tissues and normal tissues. Although HSC70 was not significantly or highly enriched in renal cancer cell lines, HSC70 was differentially expressed in papillary renal cell carcinoma. We conclude that HSC70 expression may serve as a prognostic indicator of renal cancer.

There are some limitations to our study. HSC70 may be involved in tumorigenic events, such as metastasis, invasion, and drug resistance. However, a detailed mechanism underlying the function of this protein with respect to the regulation of renal cancer progression is lacking. Further studies should be performed to explore the molecular mechanisms by which HSC70 regulates the biological behavior of renal cancer cells and tissues and to find new ways of treating patients with renal carcinoma.

## Supplementary Material

Supplementary figures.

## Figures and Tables

**Table 1:** Correlations between HSC70 expression and clinicopathological characteristics

Variables	HSC70 expression (%)	No. HSC70 expression (%)	*P*-value
No. of patients	50 (41.3)	71 (58.7)	
**Age (year)**			0.302
≤65	33 (66.0)	53 (74.6)	
>65	17 (34.0)	18 (25.4)	
**Histological grade**			0.071
I	29 (58.0)	43 (60.6)	
II	13 (26.0)	25 (35.2)	
III	8 (16.0)	3 (4.2)	
**The level of serum Ca^2+^**			0.856
Normal	21 (42.0)	31 (43.7)	
Unnormal	29 (58.0)	40 (56.3)	
**The level of Hemoglobin**			0.190
Normal	19 (38.0)	19 (26.8)	
Unnormal	31 (62.0)	52 (73.2)	
**Neutral Granulocyte Count**			0.737
Normal	21 (42.0)	32 (45.1)	
Unnormal	29 (58.0)	39 (54.9)	
**Platelet count**			0.951
Normal	48 (96.0)	68 (95.8)	
Unnormal	2 (4.0)	3 (4.2)	
**Distant Metastasis**			0.019
Yes	14 (28.0)	8 (11.3)	
No	36 (72.0)	63 (88.7)	
**Death**			0.019
Yes	13 (26.0)	7 (9.9)	
No	37 (74.0)	64 (90.1)	

## Abbreviations

HSC70: heat shock protein 70
ccRCC: clear cell renal cell carcinoma
OS: overall survival
HRP: horseradish peroxidase
