# Supplementary figures and images for: Clinical Implications of HSC70 Expression in Clear Cell Renal Cell Carcinoma
Source: Int J Med Sci. 2021 Jan 1;18(1):239–44. doi: 10.7150/ijms.43100 (PMC7738978; doi:10.7150/ijms.43100)

Supplementary figure 1

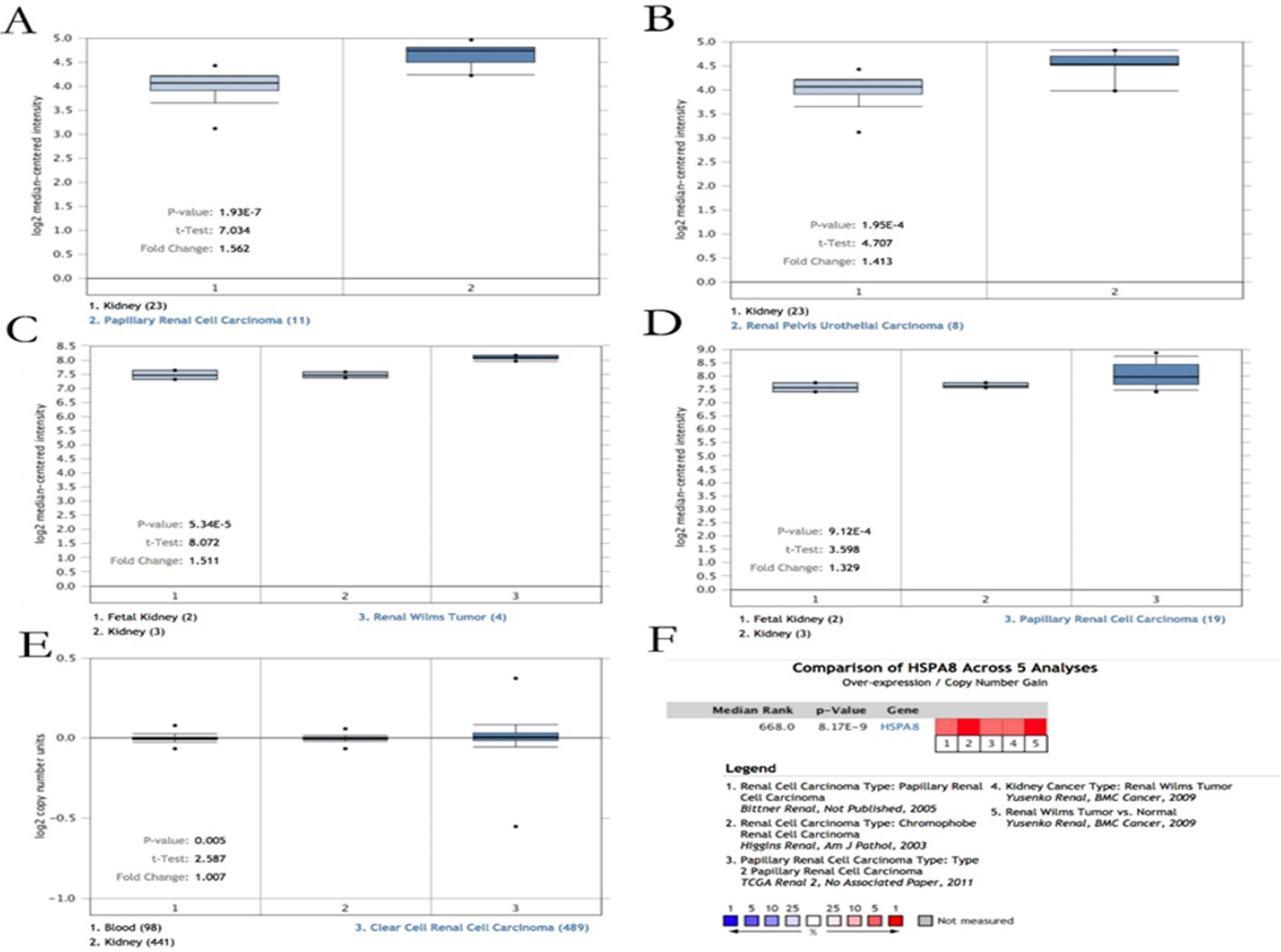

Supplementary figure 2

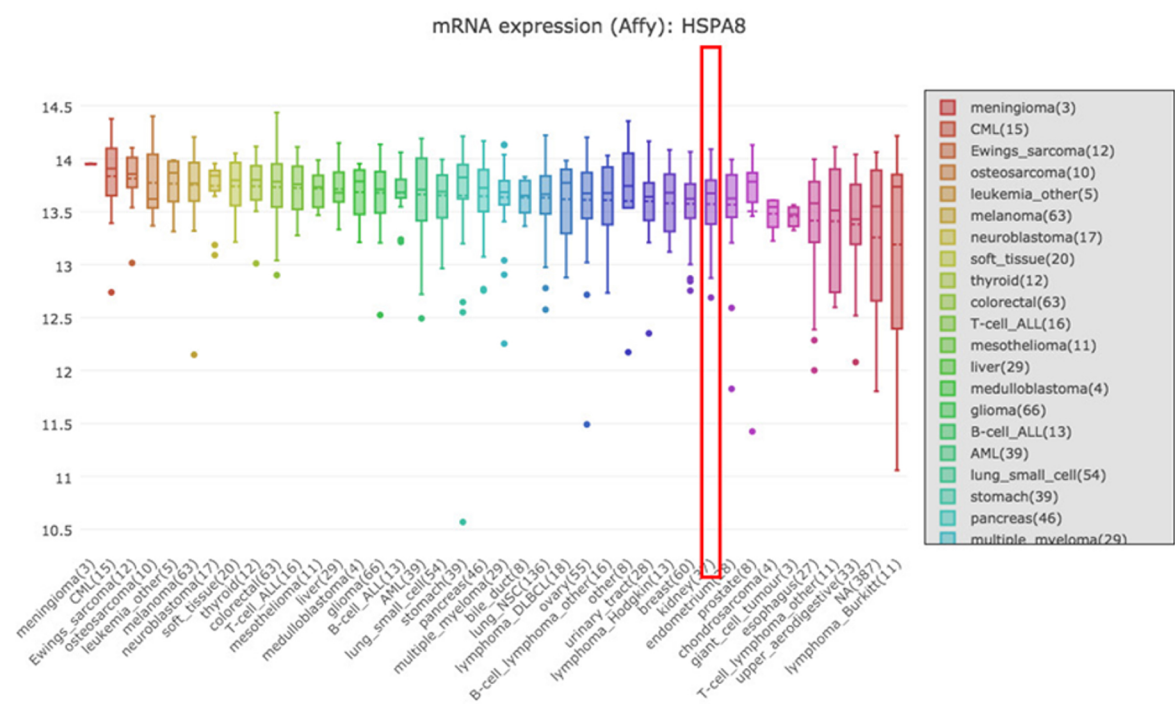

Supplement: Supplementary file 1 — Supplementary figures. [file ijmsv18p0239s1.pdf]
